# Barriers to formal healthcare utilisation among poor older people under the livelihood empowerment against poverty programme in the Atwima Nwabiagya District of Ghana

**DOI:** 10.1186/s12889-019-7437-2

**Published:** 2019-08-28

**Authors:** Williams Agyemang-Duah, Charles Peprah, Prince Peprah

**Affiliations:** 1 Department of Planning, Kwame Nkrumah University of Science and Technology, Kumasi, Ghana; 2 Natural and Built Environment, Sheffield Hallam University, Sheffield, UK

**Keywords:** Barriers, Formal healthcare, Formal healthcare utilisation, Older person, Ghana

## Abstract

**Background:**

Even though there is a growing literature on barriers to formal healthcare use among older people, little is known from the perspective of vulnerable older people in Ghana. Involving poor older people under the Livelihood Empowerment Against Poverty (LEAP) programme, this study explores barriers to formal healthcare use in the Atwima Nwabiagya District of Ghana.

**Methods:**

Interviews and focus group discussions were conducted with 30 poor older people, 15 caregivers and 15 formal healthcare providers in the Atwima Nwabiagya District of Ghana. Data were analysed using the thematic analytical framework, and presented based on an a posteriori inductive reduction approach.

**Results:**

Four main barriers to formal healthcare use were identified: physical accessibility barriers (poor transport system and poor architecture of facilities), economic barriers (low income coupled with high charges, and non-comprehensive nature of the National Health Insurance Scheme [NHIS]), social barriers (communication/language difficulties and poor family support) and unfriendly nature of healthcare environment barriers (poor attitude of healthcare providers).

**Conclusions:**

Considering these barriers, removing them would require concerted efforts and substantial financial investment by stakeholders. We argue that improvement in rural transport services, implementation of free healthcare for poor older people, strengthening of family support systems, recruitment of language translators at the health facilities and establishment of attitudinal change programmes would lessen barriers to formal healthcare use among poor older people. This study has implications for health equity and health policy framework in Ghana.

**Electronic supplementary material:**

The online version of this article (10.1186/s12889-019-7437-2) contains supplementary material, which is available to authorized users.

## Background

The number of older people aged 60 years or over worldwide is growing at 3.2% every year and will follow the same trend in the years ahead [1, 2]. In 2015, the population of older persons aged 60 years or more in the world was 900 million and is predicted to exceed 2 billion by 2050 [3]. Therefore, it is expected that the older population across the continents of the world would reach 35% in Europe, 28% in North America, 25% in Latin America and the Caribbean, 24% in Asia, 23% in Oceania and 9% in Africa by 2050 [1]. It is estimated that the population of older people aged 60 years or above in sub-Saharan Africa is expected to rise from 46 million in 2015 to 161 million by 2050 [4].

Similarly, the population of older people in Ghana has increased at a rate of 7.2% which surpasses all countries in sub-Saharan Africa [5]. This increase is attributed to the decrease in fertility rates, increasing life expectancy [2, 6–8], improved medical care [6], nutrition, healthcare education and income [5]. However, due to the increase in ageing population, it is likely that the pressure on healthcare systems will be enormous [9] because of the morbidities such as physical impairments, respiratory diseases, cognitive and functional decline, mental health conditions [10, 11], communicable and non-communicable diseases [12, 13] including hypertension, kidney disease, diabetes associated with ageing [14]. As a result of these health challenges associated with ageing, older people may demand frequent healthcare services [2].

In most cases, the utilisation of formal healthcare is viewed as receiving medical treatment from a health provider at a facility including hospitals, health centres and clinics [15]. In Ghana, whereas 51.7% of the general population utilise healthcare facilities [16], between 17.8 and 52.41% of older people respectively utilise private and public healthcare facilities [17, 18]. Factors that predict such healthcare utilisation include health insurance status [19], education, gender [16, 19], age, social status, marital status, ethnicity, religion, family size, employment and type of occupation [16]. Empirical evidence has shown that demographic, socio-economic [20, 21] and health status factors [22–24], are associated with formal healthcare utilisation among older people.

Issues such as financial problems, limited health workforce, and facilities are the major challenges facing the healthcare system in Ghana [25, 26]. Consequently, the utilisation of formal healthcare services among older people involves numerous barriers [10, 27, 28]. These barriers are mostly factors that hinder access to and utilisation of formal healthcare services [29]. Social, cultural, economic, institutional factors including health illiteracy and language difficulties [27, 29–31], geographical distance and transportation problems [10, 13, 28, 32–34], societal cultural norms [35, 36] and lack of health insurance [37] impede the use of formal healthcare among older people. Despite the global growth in literature on barriers to formal healthcare use among older people, little is known from the perspective of poor older people in Ghana.

In this study, poor older people were defined as those who are 65 years or more and are enrolled in the Livelihood Empowerment Against Poverty (LEAP) programme [38, 39]. Sponsored by the World Bank, United Nations International Children’s Emergency Fund and Government of Ghana, the LEAP programme provides a financial package to extremely poor households including older people aged 65 years or more [40, 41]. The recipients receive between GH¢ 64 and 106 (US$ 13.42–22.23 as at the period of the study) every 2 months [39]. Beneficiaries have the opportunity to determine how to spend the LEAP grant on their basic needs such as healthcare [39]. It further provides a free enrollment in the National Health Insurance Scheme (NHIS) for beneficiaries [40] with the aim of improving their healthcare utilisation [42]. Focusing on poor older persons, this work explores barriers to formal healthcare use in the Atwima Nwabiagya District of Ghana.

The focus of poor older persons under the LEAP programme enabled us to select those categorised as poor older people in the study district. This study concedes that knowing the specific factors that inhibit older people’s formal healthcare utilisation and the interaction among them in Ghana is important in ensuring that they have adequate health security and recognisable dignity to contribute to national development. This study is important because problematic access to and utilisation of formal healthcare services among poor older persons under the LEAP programme could inhibit the attainment of the United Nations' health-related Sustainable Development Goals.

## Methods

### Study setting and design

This current study used methods from our previously published works. Details of the methods have been reported elsewhere [38, 39]. Like our previously published studies, this study took place in three purposively selected rural communities (Kobeng, Amadum-Adankwame, and Offinso Adagya) and five formal healthcare facilities (Nkawie Toase Government Hospital, Afari Community Hospital, Akropong Health Centre, Dr. Frimpong Boateng Medical Centre, and Mount. Sinai Hospital) in the Atwima Nwabiagya District of Ghana. The decision to involve these various health facilities did not take cognisance of spatial discourse because, upon discussion with the respondents, it was revealed that the selected health facilities remained the main treatment centres for poor older people in the district. The district has a population of 5430 older people, with 24.36% ageing between 65 and 69 years whiles 31.7% fall within 70–74 years. Out of this number, 401 are enrolled in the LEAP programme.

Overall, the district has twenty-eight (28) health facilities with the greater part of the facilities, for example (15) 53.6% being possessed by private professionals with (12) (42.9%) having a place with Ghana Health Service (GHS) and one (1, 3.5%) being a Christian Health Association of Ghana (CHAG) facility. Within the sub-areas of the district, a total of seven (7) functional Community-Based Health Planning and Services (CHPS) compounds can be found. The main referral point for the remaining facilities is the Nkawie Toase Government Hospital, which is the district hospital.

The ethnic and cultural diversity of the district population additionally made it reasonable for conducting this research. The qualitative study approach was employed for a holistic understanding of older people’s standpoint on issues serving as hindrances to their use of formal healthcare [43, 44]. With this approach, much prominence was given to the participants’ feelings, experiences and belief systems concerning formal healthcare use barriers during the data collection process [45]. This ensured a maximum interaction and collaborative effect between the researchers and the participants throughout the data collection process as participants were given the opportunities to freely express themselves on their formal healthcare use barriers [46]. In this case, the researchers and the study participants were related and commonly intuitive and stayed open to new information during the data collection. This gave a point by point depiction of factors serving as barriers to the use of formal healthcare in the study area.

### Sampling and recruitment procedures

In this study, five including three private and two public health facilities were purposively selected (see study setting and design). This was done to get a blend of ideas, experiences and opinions on poor older people's perspective on barriers to formal healthcare use in three different communities of Kobeng, Amadum-Adankwame and Offinso Adagya. It must be emphasised that all the private healthcare facilities included in this study have signed on to the NHIS and thereby accept National Health Insurance Card to provide healthcare to enrollees. The enrolment of different healthcare facilities was additionally proper because the researchers needed to get a diversity of experiences and opinions on formal healthcare use barriers from various health stakeholders to make sound conclusions and recommendations.

The study utilised non-probability sampling techniques of purposive and convenience sampling strategies to recruit a total of 60 participants comprising 30 poor older people, 15 caregivers and 15 formal healthcare providers. It must be emphasised that the recruitment strategy was an arbitrary in nature as it did not account for population size [47] but rather informed by data saturation as no new information were coming after this respective number of participants were interviewed. Healthcare providers were purposively selected because of their in-depth knowledge on the subject matter as well as the important role they play in healthcare use [44, 48]. On the other hand, a convenience sampling technique offered the study the flexibility to select specific respondents like the caregivers and poor older people based on their availability and readiness [38, 39, 49, 50].

### Data collection instrument and procedure

As the study deals with opinions, experiences and feelings, in-depth interviews and focus group discussions (FGDs) were conducted to obtain data for the study [43, 44, 51, 52]. This enabled the researchers to obtain a deeper understanding of the topic under investigation by probing the study participants in several ways. A total number of 60 interviews were conducted to elicit data for the study. During the interviews, the respondents were granted the freedom to express their opinion concerning the events, behaviours, and beliefs regarding the objective of the study [53]. Where further clarification was needed, respondents were probed and this helped the researchers to get the needed information [53]. All the three categories of respondents (poor older people, healthcare providers and caregivers) took part in the interview. The questions, basically, focused on background information such as gender, education, religion, ethnicity and the barriers they encounter in their quest to use formal healthcare services. Interviews with poor older people and caregivers took place at their various homes which provided a friendly and relaxed environment devoid of fear and suspicion for the interaction [54]. Concerning the formal healthcare providers, interviews were conducted after their daily work schedules mostly in free consulting rooms at their respective health facilities. Interviews with the caregivers and the poor older people were for a duration of 40–50 min whereas that of the  healthcare practitioners lasted 45–60 min. All the interviews were captured through audio-recording with the participants’ consent, and handwritten field notes were also made.

On the other hand, the FGDs were done for only poor older people. The FGD guide used was specifically developed for this study (see Additional file 1). The FGDs enabled the participants to talk more openly and freely because they share the same background or experience. The FGDs took place at classrooms and churches that were free from third party interference. Each group discussion comprised 8–10 participants and lasted approximately between 90 and 100 min and ended at a point where the researchers felt all issues have been covered. In all, three FGDs were done, one in each of the selected study communities. According to Bhattacherjee [48], in FGDs, the interaction is led by a person with adequate knowledge on the subject matter to guarantee a better understanding of the issue by the group members. The role of the moderator is to facilitate the discussion rather than lead the discussion [44]. The discussions primarily focused on formal healthcare use barriers among poor older people.

The interviews were mostly conducted in ‘Twi’ which is the local language of the respondents with few instances in English to satisfy the preferences of the interviewees. Also, with the informed consent of the participants, discussions at the group meetings were audio-recorded whiles handwritten field notes were further made [44].

### Trustworthiness

In this study, we emphasised on trustworthiness by maintaining and ensuring credibility, transferability, conformability and dependability throughout the study especially during the data collection process. Practical trustworthiness steps included the use of purposive and convenience sampling strategies, member checks, lengthy interactions with the participants and expert review of transcripts. Again, the researchers shared summaries of the findings with interested study participants to ensure that the results reflect their expressed views and opinions.

### Ethics approval and consent to participate

The Committee on Human Research Publication and Ethics (CHRPE), School of Medical Sciences, Kwame Nkrumah University of Science and Technology and Komfo Anokye Teaching Hospital, Kumasi, Ghana granted the ethical approval for this study (Ref: CHRPE/AP/311/18). Furthermore, participants were briefed on the purpose of the study and informed consents were obtained from interested participants. Participants were again assured of anonymity and confidentiality of their expressed opinions. Participation in the study was completely voluntary and participants were at liberty to stop participating whenever they wished to do so.

### Data analysis

All the recorded responses that were not in English were translated into English. The transcripts were cross-checked back-to-back with the original audio responses and written notes to obtain accurate, quality and reliable data for the study. The transcripts and field notes were read and reviewed several times by the authors with the objective of identifying related trends and differences in the responses. Through an a posteriori inductive method, the authors developed broad and consistent themes, based on the participants’ true experiences and feelings [55]. The thematic analytical approach helped the researchers to identify, analyse and report patterns within data whiles aiding in the organisation and description of the data in rich detail [56]. The study findings were therefore presented according to the main and sub themes that emerged from the analysis and some interesting expressive views of the participants were quoted to support the narration and description.

## Results

### Background characteristics of participants

In all, 60 participants comprising 30 poor older people (users), 15 formal healthcare providers and 15 caregivers took part in this study. With regard to users, most (23) of them were females, had no level of education (19), Christians (27) and Akan (25). Regarding the formal healthcare providers, nine were females, 12 each were Christians and Akan and all had attained a tertiary level of education. Concerning the caregivers, all of them were females (15), eight had no level of education, 14 were Christians and 13 were of Akan ethnicity (Table 1).

**Table 1:** Sample characteristics of the study participants

		Category of Respondents (*N* = 60)		
Variable	Category	Users (poor older people) (*N* = 30)	Providers(*N* = 15)	Caregivers(N = 15)
Gender	Male	7	6	–
	Female	23	9	15
Education	None	19	–	8
	Basic	8	–	5
	Secondary	3	–	2
	Tertiary	–	15	–
Religion	Christianity	27	12	14
	Islam	3	3	1
Ethnicity	Akan	25	12	13
	Northerner	5	3	2

### Barriers to formal healthcare utilisation

The results covered the opinions of all the study participants such as poor older persons, caregivers and formal healthcare providers. The results were further categorised into theme clusters. The four main barriers were physical accessibility barriers (poor transport system and poor architecture of facilities), economic barriers (low income coupled with high charges and non-comprehensive nature of the NHIS), social barriers (communication difficulties and poor family support) and unfriendly nature of healthcare environment barriers (poor attitude of providers) (Table 2).

**Table 2:** Main themes and associated sub-themes

Main themes	Sub-themes
Physical accessibility barriers	• Transportation- high transport cost and bad nature of roads • Poor architecture of facilities
Economic Barriers	• Low income coupled with high charges • Non-comprehensive nature of the NHIS
Social barriers	• Communication/language issues • Poor family support
Unfriendly nature of healthcare environment barriers	• Poor attitude of healthcare providers

### Physical accessibility barriers

#### Transportation- in terms of cost and bad road network

Physically, most of the poor older people are not required to travel more distance for healthcare due to their health conditions. As a result of distance and transportation problems, poor older people in more remote areas have higher difficulties of access to medical care. These problems become more critical for those specifically poor older persons since they cannot easily walk to health centers. Participants emphasised that in the rural areas, most health facilities are situated at the capitals and other few towns in the study area whilst the roads linking people to these areas are deplorable. The study participants stressed that road networks often create an accessibility challenge by serving as a barrier:

*“I think poor road networks, especially in rural and remote areas is also a barrier to healthcare utilisation among the older people in Ghana. The roads we have to use before getting to the nearest health centres are in a bad state. The hassles that we pass through before reaching a health centre are serious. The poor road networks serve as a barrier, and where there is a barrier, there is a utilisation challenge.”*(A 72-year old poor older person, FGD)

They expressed that bad roads prevent people from using formal healthcare services. They established that though health facilities may be in most rural and other areas in Ghana, the roads linking them to the users are not good hence, serving as a barrier to formal healthcare utilisation.

*“My concern is about the nature of the roads that link us to various health centres. In fact, most of the roads are too bad to be used by the poor older people. This has created serious utilisation problems. In some areas, health centres are available but utilisation is a problem due to bad roads linking to these health centres.”*(A 31-year old caregiver, Interview)

The poor road network has resulted in higher transport charges which the poor older people described as unbearable. The poor older people maintained that because of the bad roads many drivers in most cases refuse to use them, especially in rainy seasons when people have to pay huge amount of money for a shorter distance before accessing healthcare.

*"Because the road is bad, drivers most often refuse to take us to the town where the health centre is located and those who accept to go charge higher fares. At times such charges are unbearable for us so we decide not to go at all."* (A 69-year old poor older person, Interview)

Another poor older person from Kobeng said:

*“High cost of transportation. I spend a lot of money on transportation. I have to hire a car before I can go to the hospital. The poor nature of the road does not allow many commercial vehicles to come to the village. Those few ones that come charge us heavily before they come. The difficulty I go through before getting to the hospital is too much for me. Authorities should take a second look at our road else we cannot use healthcare.”* (A 65-year old poor older person, FGD)

One caregiver from Amadum-Adankwame summarised the discussion on transportation:

*“The road is bad. Drivers are refusing to come here because of the poor nature of the road. The government should reshape the road for us in order to reduce the cost of transportation so that we can use healthcare on time. If the road is good, less money will be needed in terms of transportation. Again drivers would be willing to bring their cars to this community. Even at night when you call for a driver to pick you up to the hospital, it will not be a problem.”* (A 40-year old caregiver, Interview)

#### Poor architecture of facilities

The study participants stated that most healthcare facilities in the study area are not user-friendly for the poor older people. This is because the facilities do not have any laid down assistance or special care for the poor older people. The poor older people particularly mentioned that no healthcare facility in their vicinity has a system in place whereby the poor older people are physically assisted in terms of walking or moving from one consulting room to the other.

Aside from the perceived absence of these arrangements, physical barriers in the form of poorly designed buildings were mentioned by the poor older persons as a barrier to their formal healthcare service utilisation. Considering the frailness of most of the poor older people, they preferred not to climb staircase before utilising healthcare. However, some of the health facilities in their catchment area are located on the second and third floor of buildings.

One poor older person complained:

*"I mostly do not go to the hospital because I suffer a lot when I go. This is because they have no support in place to assist the poor older people in terms of walking. One thing that worsens the case is that they are located on the second and third floors which make climbing very difficult for us. Look at my health condition and imagine me climbing a staircase, how do you think it would look like?"* (A 75-year old poor older person, FGD)

### Economic barriers

#### Low income coupled with high charges

Interestingly, despite all the study participants receiving grants from the LEAP programme in every 2 months, financial problems were disclosed to be the most pressing barrier to formal healthcare utilisation. Both poor older people and healthcare providers explained that the limited grants received from the LEAP coupled with the cost involved in using formal healthcare, which is very high, do not allow most of the poor older persons to afford formal healthcare. The financial challenge of the poor older people mostly stemmed from their inability to work for income. The evidence from the interviews and FGDs confirmed the status of older persons as poor in terms of income and as a result unable to pay for any health services involving higher charges. It was interesting to find people receiving grants identifying financial challenge as their main obstacle to formal healthcare utilisation. The LEAP grant which is perceived to be insufficient was used for food, clothing, and shelter, among others by most poor older people. After spending on these basic needs, the remaining amount of the grant becomes inadequate for accessing formal healthcare services. It was found that some poor older people borrow before they are able to access formal healthcare and repay when the LEAP grant comes. This act of borrowing has, therefore, become a coping strategy for most of the poor older persons in terms of accessing formal healthcare.

Meanwhile, almost all poor older persons were willing to use formal healthcare services, but poverty and high healthcare charges served as obstacles to their use of formal healthcare services.

One female poor older person from Kobeng complained:

*"Financial problem is killing us because without money you cannot acquire the required drugs and treatment. The doctor has told me to visit the hospital every two weeks for a check-up but I am unable to adhere to this because of financial problem. As we are speaking, I am supposed to go to the hospital, but I couldn't go because of a lack of money. The LEAP money, on the other hand, is too low to cater for my basic needs including health whereas the hospital's charges are also high. In fact, access to regular healthcare service use is very difficult for us due to a financial problem." (*A 66-year old poor older person, FGD)

Another caregiver from Kobeng lamented:

*“The last time I took my mother to the hospital, I needed to borrow before I was able to send her. Due to lack of money, I always have to delay in seeking healthcare for my mother who is an older person. When they prescribe drugs, we are unable to get money to buy, this is making it difficult for us in terms of healthcare utilisation”.* (A 44-year old caregiver, FGD)

 A provider from Nkawie Toase Government Hospital concluded:

*“Personally, I can say from my experience as a senior nurse that finance is the main problem facing the poor older people in terms of accessing healthcare service. Most of them are economically handicap and so cannot get money to pay their medical bills and purchase drugs especially those that are not covered under the health insurance. Mostly, they do not adhere to treatment and check-up schedules, mainly due to lack of money. At times, we have to give them money for transport back home.” (A* 43-year old healthcare provider, Interview)

All these findings attest to the fact that financial status of the poor older people is key in their access to formal healthcare. Thus, eliminating financial barriers to accessing formal healthcare amongst low socio-economic groups may have a positive effect on formal healthcare utilisation.

#### Non-comprehensive nature of the NHIS

The study participants admitted that the introduction of the national health insurance scheme has had a positive impact on formal healthcare utilisation among poor older persons. They mentioned that the health insurance card serves as a facilitator of formal healthcare utilisation among the poor older people. However, due to the non-comprehensive nature of the insurance scheme, the card in some cases acts as a barrier to formal healthcare utilisation for many holders. Most of the poor older people were having active health insurance cards, however, the cost incurred at the facilities often exceed what the insurance could pay for and as a result required to pay for the additional charges. Few poor older persons who were not having valid or active health insurance were therefore required to pay the full bill whenever they visited the hospital and those who were unable to pay were prevented from accessing formal healthcare. All the participants explained that insurance does not cover most healthcare costs, especially expensive drugs and serious medical interventions such as surgeries.

One older person from Amadum-Adankwame had this to say:

*“I think health insurance is another barrier. This is because even if you hold an active insurance card it does not cover all medical bills especially the expensive drugs and surgeries. Our medical bills are often higher because of the disease we battle with such as hypertension, diabetes, stroke, among others. So as the insurance does not cover the cost of treatment of these diseases it becomes difficult for us to use formal healthcare services even with the card.”* (A 69-year old poor older person, FGD)

One provider from Dr. Frimpong Boateng Medical Centre agreed with this opinion:

*"Few of poor older people are not covered under the National Health Insurance Scheme. Those with health insurance should also have to do some top up in most cases and this I think at times prevents some of the poor older people from using formal healthcare. Health insurance does not cover most of the drugs so the poor older people have to buy them at their own cost. Especially with the diabetics, when you come and you are admitted to this ward, health insurance covers the first test but with the subsequent ones, the client will have pay. So for me, I think the poor older people do not need health insurance, but they rather need free healthcare."* (A 52-year old healthcare provider, Interview)

These views suggest that although health insurance in itself is good in terms of facilitating formal healthcare utilisation among the poor older people, the non-comprehensive nature of it in some instances makes it a barrier to formal healthcare utilisation. This is because poor older people will have to incur extra cost before utilising healthcare fully, especially those with severe health needs.

### Social barriers

#### Communication/language issues

Most poor older people, as well as healthcare providers, indicated language as a barrier to formal healthcare utilisation. On the part of poor older people, most providers cannot speak the local dialect (Twi) while the users do not also understand/speak English hindering effective communication between the two parties.

A poor older person from Amadum-Adankwame had this to say

*“We find it difficult to explain to the doctors and nurses our health conditions, especially when the provider is non-native or non-speaker. Most of the providers in many health facilities cannot speak the local language (Twi) fluently, whereas we cannot also communicate with them in the English language.”* (A 67-year old poor older person, Interview)

Another poor older person from Amadum-Adankwame commented:

*“Aside from financial problem, language also prevents some of the poor older people from using formal healthcare services in this community. This is because most of the professionals do not understand our local dialect and we also do not understand English either. We should try and encourage our nurses and doctors to learn the local dialect or those who can speak the local dialect should be allowed to work in this community.”* (A 65-year old poor older person, FGD)

A provider from Afari Community Hospital endorsed this view:

“*I share the view that language is another barrier to formal healthcare utilisation among the poor older people in this community. From my experience as a health worker for several years, I have observed that some of the poor older people who come to the hospital are mostly unable to speak the English language which affects effective communication between them and us, especially when the health assistants do not comprehend the local language either.” ( A* 37-year old healthcare provider, Interview)

#### Poor family support

Looking at the physical and health conditions of most poor older people, they will require assistance in terms of seeking healthcare. The majority of the poor older people needed someone to assist them before they could either walk or board a vehicle to the hospital. At the same time, at the health facility, the poor older people would again need someone who will do the errand. Some of the poor older persons mentioned that in some instances there is no one to accompany them to a health facility. As such, they are unable to use healthcare services despite having money to fund healthcare utilisation. The health providers stressed that it is always helpful for someone to follow the poor older people to health facilities. This is because, in most cases, some of the poor older individuals find it difficult to walk, explain their health conditions and adhere to treatment. Healthcare providers further maintained that poor older people with caregivers use formal healthcare services more than those without caregivers.

*“At times I wish to go to the hospital, but I cannot go because I have no one to assist me to walk or even run the errand at the hospital. The last time I went to the hospital, I was stranded as I had no one to talk to the nurses on my behalf. So, I remember one of the nurses told me to come with someone whenever I am coming to the hospital. From there, I have not gone to the hospital again because I still have no one to go with me and I do not want to become stranded again, if I have someone to assist, I will go.”* (A 68-year old poor older person, Interview)

A poor older person from Offinso Adagya also added:

*“My problem is someone who will assist me to walk to the health facility and also attend to the calls of the health workers. At my age, it is always frustrating to go to the hospital without someone escorting you. The nurses would be calling you here and there and you would need someone who will be attending such calls. Also, I have irretentive memory so I would need someone who will listen to drug prescriptions and instructions on my behalf. So, at times I do not go to the hospital if I have no one to accompany me.”* (A 78-year old poor older person, FGD)

One health provider Akropong Health Centre also shared a similar view:

*“I think lack of caregivers is also another form of barrier to healthcare utilisation among the poor older people. At times, when they fail to come for a check-up on a scheduled date, one of the reasons they mention aside income is lack of caregiver who will bring them him to the hospital since they are unable to walk without assistance. From experience, the poor older people with caregivers use healthcare frequently than those without caregivers”* (A 33-year old healthcare provider, Interview)

### Unfriendly nature of the healthcare environment

#### Poor attitude of healthcare providers

In the utilisation of formal healthcare, the attitude of providers plays a significant role globally. Attitude stems from providers’ professionalism, confidentiality, treatment, and interpersonal relationship. Almost all the poor older people interviewed mentioned perceived poor attitudes of health workers, especially nurses as a factor inhibiting formal healthcare utilisation. Some mentioned the poor human relationship between them and the healthcare providers as a barrier. They mentioned that the unfriendly and unapproachable nature of most nurses in formal healthcare centres, especially those at public health facilities influences their decision not to use formal healthcare. Looking at the health and physical conditions of most of the poor older people, sensitivity, care, and attention would be required from health workers, however; the poor older people maintained that these are mostly not found among health workers, especially the nurses at public health facilities. This is what a participant from Kobeng said:

*"Disrespect on the part of some nurses is another thing which is preventing me from using formal health, especially the public ones. Some are very uncordial and disrespectful. They do not have time for poor older people. Some of the nurses should be talked to because a smile from a nurse is a source of medicine".* (A 70-year old poor older person, FGD)

A caregiver from Offinso Adagya further complained:

*“When I took my father to the hospital this was what the nurse had to say ‘you are fortunate your father is old; others fathers’ don't come close to your father's age. Stop disturbing me'.*” (A 39-year old caregiver, Interview)

Another poor older person from Amadum-Adankwame in addition criticised:

*“Disrespect on the part of nurses’ most especially female nurses are common in the government hospital which mostly prevents me from attending to the hospital. It is not common in private hospitals because when the patient report, you could be sacked instantly, but this is not common at the government hospital making some nurses to behave that way. I went to the hospital and complained about waist pains. The best the nurse could do was to embarrass me. ‘Go away there is no drug for waist pains. Haven’t I told you?’*” (A 77-year old poor older person, FGD)

## Discussion

The specific barriers to formal health services use among poor older people receiving LEAP grants have not been explored. The collection of qualitative data from three important local stakeholder groups (poor older people who are 65 years or above, caregivers and formal healthcare providers) made an analysis of this important area of inquiry possible. To the best of the authors’ knowledge, this is the first study to explore and document an in-depth understanding of the various formal health services use barriers among poor older people receiving financial support from the LEAP programme. This study is therefore unique in its contribution of valid and reliable evidence on formal health service use barriers among poor older people. The main barriers identified were: i) physical accessibility barriers including poor transport system and poor architecture of facilities ii) economic barriers comprising low income, high charges and non-comprehensive nature of the NHIS iii) social barriers such as communication/language and poor family support and iv) unfriendly nature of the healthcare environment including poor attitude of healthcare providers. This confirms that poor older people experience multiple barriers to accessing formal health services in Ghana. Clearly, the formal health services use barriers among poor older people found in the present study do mirror the barriers mentioned in the literature [27, 32, 37–39, 57–59].

From the findings, it is clear that the barriers involved in using formal healthcare services among poor older persons under the LEAP programme begin right at home, especially during the period of deciding and contemplating on where to get money for bills, transport service to the health facility, who would support them to the health facility, how the providers would treat them and how to communicate their health conditions to the provider, among others. Despite all poor older people receiving financial support from the LEAP programme in every 2 months, they are unable to pay for their health services at formal healthcare facilities. The insufficiency, irregular payment mode and diverse health needs of the poor older people under the LEAP programme could partly explain their financial challenge [38, 39, 41]. The cost involved in treating most of the diseases among poor older people in this study was considered high and the LEAP grants  alone was inadequate since it is not for only health needs but other basic needs such as food [38, 39]. Unfortunately, the health insurance which is to lessen the financial burden of poor older people under the LEAP programme [60] in a way serving as a barrier to formal healthcare utilisation among poor older people due to the non-comprehensive nature of the scheme [29]. As the insurance does not cover all the medical bills, especially treatment with higher charges, the poor older people under the LEAP are required to pay for some portions of their medical bills and those with no such amount to pay are mostly not able to use healthcare services. This means that eliminating financial barriers to accessing formal healthcare amongst poor older people especially those under the LEAP programme in Ghana may have a positive effect on formal healthcare utilisation [38, 39].

Transportation in terms of cost of transport service and bad road networks also constituted an obstacle to formal healthcare utilisation among poor older people under the LEAP programme in Ghana. Due to the poor nature of roads linking them to formal health facilities, which are mostly located at the capitals and other big towns, transport services are inadequate and highly expensive [25, 61]. Meanwhile, due to the limited physical strength of most poor older people under the LEAP programme, walking to the hospital is much more difficult. Without transportation, even a shorter distance to care can become an insurmountable problem. The opportunity for poor older people to have a vehicle to transport them to a practitioner or facility is especially important in rural settings of Ghana where distances to health facilities are relatively high with poor road quality, and public transportation is seldom available [28, 62].

Moreover, language differences and poor family support have featured in many healthcare studies among poor older people in most African countries [28–30, 38, 61, 63]. The inability of the health providers to communicate in the local language of older people affects the healthcare process and system [29]. Conversely, poor older people are also unable to communicate how they feel or the symptoms of the diseases to the health providers. This scenario presents a difficult task for the poor older people to express themselves and the providers to understand them [29, 30]. This often results in the decision to stay away from using formal healthcare on the part of poor older people even if they need it. Other studies have reported similar findings. For instance, in Namibia, language differences were found to be a key barrier to healthcare utilisation among older people accessing formal healthcare [28, 64]. Specifically, in Van Rooy et al.’s [28] study poor older people complained that health providers address them using English (considered a foreign language) which hinders effective communication between them because of their limited English literacy [29, 63]. Regarding this, the presence of translators at the facilities which is the standard internationally could help promote effective communication between users and providers [29]. Meanwhile, the practice of older people accompanied to healthcare facilities by caregivers who have at least some proficiency in the English language is greatly encouraged.

In addition, perceived non-respectful attitude and unapproachable interaction style of most formal healthcare providers are considered as a barrier to formal healthcare utilisation among poor older people under the LEAP programme. Poor older people perceived most of the providers to be not responsive, respectful and sensitive. In one study, it was noted that older people expected sensitivity rather than extensive medication from health professionals [10]. Considering their age and physical conditions, poor older people under the LEAP programme expect care and respect from providers, however, they mostly become disappointed because their expectations in most cases are not met. Failure to be accorded with the needed respect and care, they decide to stay away from formal healthcare utilisation. This finding confirms previous empirical findings in both developed and developing countries. For example, in the US, the most common barrier reported was the doctors’ lack of responsiveness to concerns, cited by almost one-third of respondents (32.9%) [59]. Likewise, in Namibia, poor provider attitudes were reported by older people [28]. Also, Aboderin and Beard [58] reported that older patients did not use commercial providers because of the unavailability, perceived poor quality, or age insensitivity of services in government facilities. These findings suggest that a change in providers’ attitude may improve formal healthcare utilisation among poor older people in Ghana.

Compounding the preceding discussed barriers, poor older people under the LEAP programme encounter an additional barrier in the form of poorly designed healthcare buildings. Most healthcare facilities layouts are considered by poor older people as unfriendly since they are required to climb a number of stairs at the facilities. This, to the poor older people, in some cases worsens their physical conditions.

We comment on the strengths of this study. To the best of our knowledge, this is one of the first studies in Ghana to explore barriers to the use of formal healthcare among poor older people under the LEAP programme in Ghana. This study has implication towards the realisation of the United Nations' health-related Sustainable Development Goals. Apart from this, the results from this study could guide in the design and formulation of policies that seek to address barriers to formal healthcare use among poor older people in Ghana. Some limitations were, however, notable. As a result of the use of non-probability sampling techniques, the findings must be interpreted with caution. Also, we were not able to perform an analysis on socio-demographic and health factors influencing barriers to formal healthcare use among poor older people. Additional rigorous study is required to throw more light on this association.

## Conclusion

Focusing on poor older people under the LEAP programme in Ghana, this study found barriers to formal healthcare utilisation to be related to physical accessibility, economic, social factors, and the healthcare environment. These barriers if not addressed could negatively affect their formal healthcare utilisation patterns and their human rights. We argue that improvement in rural transport services and designing of health facilities that are user-friendly for older people would be useful measures to lessen physical accessibility barriers to formal healthcare use. Implementation of free healthcare for poor older people, the inclusion of most disease burden of poor older people in the NHIS and upwards adjustment of the LEAP grants would counter economic barriers to formal healthcare use. Also, strengthening of family support systems and recruitment of language translators at the health facilities would help to counter social barriers to formal healthcare use. Attitudinal change programmes and activities such as regular orientations, sensitisation, strict monitoring and supervision of attitude of healthcare staff would aid in addressing the unfriendly nature of healthcare environment barriers particularly, poor attitude of healthcare providers. The study has implications for health equity and health policy framework in Ghana.

## Additional file

Additional file 1: FGD Guide (DOCX 14 kb)

## Abbreviations

CHAG: Christian Health Association of Ghana
CHPS: Community-Based Health Planning Services
CHRPE: Committee on Human Research and Publication Ethics
FGDs: Focus Group Discussions
LEAP: Livelihood Empowerment Against Poverty
NHIS: National Health Insurance Scheme
UNICEF: United Nations International Children’s Emergency Fund
