# Supplementary material for: Barriers to formal healthcare utilisation among poor older people under the livelihood empowerment against poverty programme in the Atwima Nwabiagya District of Ghana
Source: BMC Public Health. 2019 Aug 28;19:1185. doi: 10.1186/s12889-019-7437-2 (PMC6714403; doi:10.1186/s12889-019-7437-2)
Supplement: Supplementary file 1 — FGD Guide (DOCX 14 kb) [file 12889_2019_7437_MOESM1_ESM.docx]

**FOCUS GROUP DISCUSSION GUIDE ON BARRIERS TO FORMAL HEALTHCARE USE AMONG POOR OLDER PEOPLE**

1. Do you face any barrier(s) in using formal healthcare services? If yes, kindly share with me, the barriers you encounter in your quest to use formal healthcare services? [Probing is allowed]
2. Would you say these barriers have interfered with your use of formal healthcare services? If yes, how have these barriers interfered with your use of health care?[ Probing is allowed]
3. In general, what would you suggest must be done to improve formal healthcare use?[ Probing is allowed]
